# Scenarios of polaron-involved molecular adsorption on reduced TiO_2_(110) surfaces

**DOI:** 10.1038/s41598-017-06557-6

**Published:** 2017-07-21

**Authors:** Yunjun Cao, Min Yu, Shandong Qi, Shiming Huang, Tingting Wang, Mingchun Xu, Shujun Hu, Shishen Yan

**Affiliations:** School of Physics, State Key Laboratory of Crystal Materials, Shandong University, 27 Shanda Nanlu, Jinan, Shandong 250100 P. R. China

## Abstract

The polaron introduced by the oxygen vacancy (Vo) dominates many surface adsorption processes and chemical reactions on reduced oxide surfaces. Based on IR spectra and DFT calculations of NO and CO adsorption, we gave two scenarios of polaron-involved molecular adsorption on reduced TiO_2_(110) surfaces. For NO adsorption, the subsurface polaron electron transfers to a Ti:3d-NO:2p hybrid orbital mainly on NO, leading to the large redshifts of vibration frequencies of NO. For CO adsorption, the polaron only transfers to a Ti:3d state of the surface Ti_5c_ cation underneath CO, and thus only a weak shift of vibration frequency of CO was observed. These scenarios are determined by the energy-level matching between the polaron state and the LUMO of adsorbed molecules, which plays a crucial role in polaron-adsorbate interaction and related catalytic reactions on reduced oxide surfaces.

## Introduction

The surface oxygen vacancy (Vo) of metal oxides usually shows significant influences on the molecular adsorption and reactivity on reduced oxide surfaces^1–3^. It can serve as the preferential adsorption site of many molecules, such as O_2_^4, 5^, H_2_O^6^, alcohols^7^, and carboxylic acids^7^. Moreover, it directly promotes surface reactions such as the dissociation of alcohols and H_2_O^8–10^, the oxidation of CO over TiO_2_^1^, the coupling and enolization of acetaldehyde on CeO_2_^11^, and the aerobic coupling of amines over defective WO_3_ nanosheet^12^. Surprisingly, Vo also prohibits some photocatalytic reactions, e.g., photooxidation of trimethyl acetate^13^ and formaldehyde^14^ on TiO_2_(110) surfaces. It is generally thought that the excess electrons introduced by Vo play a crucial role in the modification of the surface chemical properties of metal oxides^15, 16^. These excess electrons usually induce the lattice distortion (polarization) in the metal oxides by Coulomb interaction. Such an electron together with its induced polarization in the oxide crystal forms a quasiparticle called a polaron. The polarons are divided into small and large polarons depending on the dimension of the lattice distortion region. For the bare reduced rutile TiO_2_(110) surface, both recent scanning tunneling microscopy (STM) and infrared spectroscopy (IR) studies confirmed that the excess electrons belong to small polarons^17–19^. That is, the lattice distortion region is of the order of the lattice constant (usually one unit cell), representing the strong interaction between electrons and phonons^20^. The resonant photoelectron diffraction (RPED) measurement revealed that these small polarons preferentially distribute in the subsurface^21, 22^.

On adsorbate-covered oxide surfaces, the polaron-involved molecular adsorption is the initial step of many chemical reactions and is important in understanding the surface chemical reactivity^15, 16, 23^. O_2_ was the most investigated probe molecule in polaron-involved molecular adsorption studies^16^ as the “electron scavenger” in photo-oxidation reactions. The O_2_ with different oxidation states plays different roles in CO oxidation and various photooxidation reactions involving organic molecules on TiO_2_ under anhydrous conditions^24, 25^, though the extent of electron transfer between O_2_ and TiO_2_ is still an open issue^26^. On rutile TiO_2_(110) surfaces, electron energy loss spectroscopy (EELS) studies^26^ showed that upon O_2_ adsorption the electronic Ti^3+^ defect state disappears and is replaced by a new energy loss at ~2.8 eV which is assigned to O_2_^−^. This further confirmed the O_2_^−^ formation during O_2_ reduction on polycrystalline TiO_2_ detected by electron paramagnetic resonance (EPR) earlier^27^. On the other hand, Petrik *et al*. suggested that O_2_ chemisorbs on TiO_2_(110) either in O_2_^2−^ or in O_2_^−^ state depending on the oxygen coverage^28^. Deskins *et al*. proposed that for the surface/adsorbate complex significant charge transfer from the reduced surface to the absorbate will occur if the electronegativity of the adsorbate is greater than the surface electronegativity^29^.

To better understand the microscopic mechanisms governing the interaction of polarons and adsorbates, the experiments using more probe molecules are urgently required. Besides O_2_ mentioned above, CO and NO are also important probe molecules. CO is the most frequently-used probe molecule whose adsorption behavior is often used to characterize the physical and chemical properties of oxide powder and single crystal surfaces^23, 30^. On TiO_2_(110) surfaces, STM study by Zhao *et al*.^31^ revealed that the preferentially adsorbed sites of CO are the next-nearest Ti_5c_ sites of Vo rather than Vo sites themselves due to the delocalized distribution of polarons. In contrast to CO, NO molecule possesses an unpaired electron in its 2π* orbital, which leads to complicated coordination chemistry on oxide surfaces^23, 32^. Recent STM studies revealed that the surface hydroxyls result in an electrostatic stabilization of the adsorbates and significantly enhance the binding energy of NO^33^, which was also supported by the density functional theory (DFT) calculations^33, 34^. The contrast study on NO and CO adsorption will give more clear scenarios of polaron-involved molecular adsorption on single crystal oxide surfaces. We expect to throw new light on this important issue by probing the subtle interaction between CO/NO and polarons through detecting the vibration frequencies of adsorbed molecules by the highly sensitive ultra-high vacuum - Fourier transform infrared spectroscopy (UHV-FTIRS)^35, 36^.

At present, we studied NO and CO adsorption on reduced TiO_2_(110) surfaces by using UHV-FTIRS and DFT calculations, and found out two typical scenarios of polaron-involved molecular adsorption on TiO_2_(110). For NO adsorption, the subsurface polaron transfers to a Ti:3d-NO:2p hybrid orbital causing the large redshift of vibration frequency (VF) of NO. While for CO adsorption, the polaron only transfers to the surface Ti_5c_ cation underneath CO, inducing a weak shift of VF of CO.

## Results and Discussion

**Figure 1:**
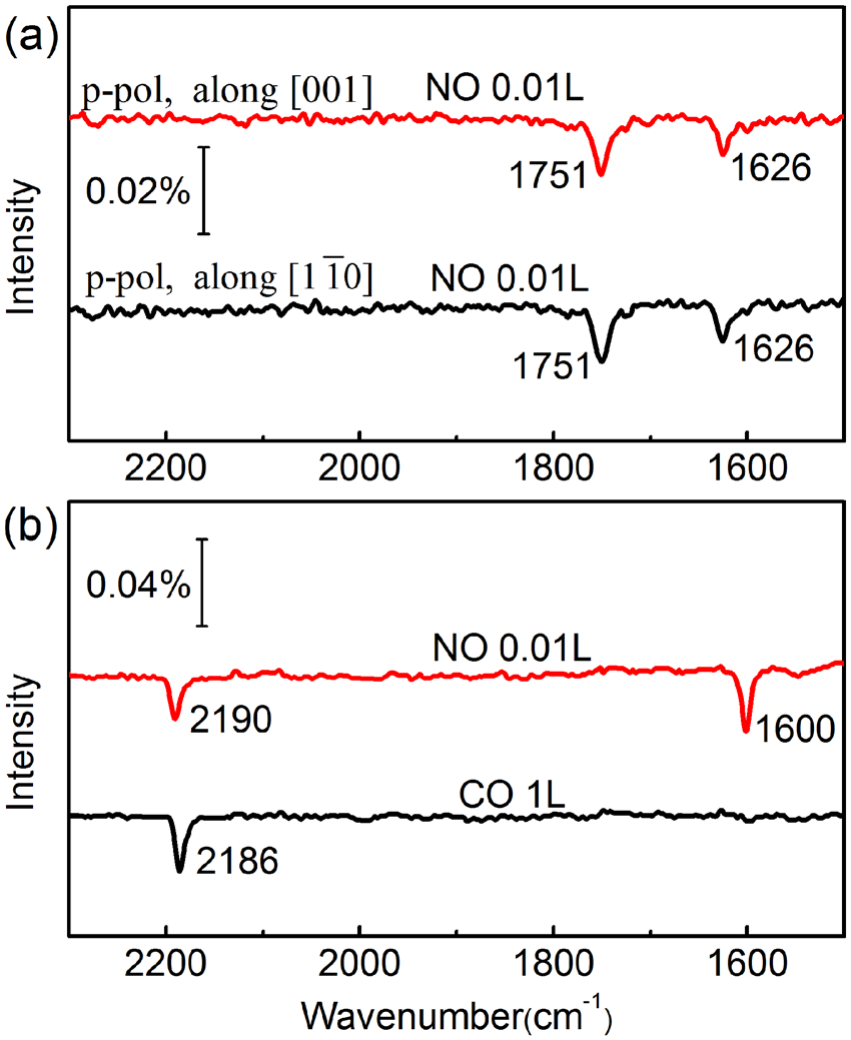
(**a**) P-polarized IRRA spectra of 0.01 L NO adsorbed on reduced TiO_2_(110) surface at 90 K. The incidence plane is along $[1\bar{1}0]$ (black curve) and [001] (red curve) direction respectively. (**b**) P-polarized IRRA spectra of NO-CO co-adsorption on reduced TiO_2_(110) surface at 90 K: First exposure 1 L CO to the reduced TiO_2_(110) surface, then 0.01 L NO.

Figure 1a shows the infrared reflection absorption spectroscopy (IRRAS) data of very low dosage of 0.01 L NO on the reduced TiO_2_(110) surface at 90 K. For the p-polarized incidence plane along both [001] and $[1\bar{1}0]$ directions, two negative IR absorption bands of adsorbed NO were observed at 1751 and 1626 cm^−1^, respectively, which are largely redshifted relative to that (1875 cm^−1^) on perfect TiO_2_(110) surfaces. Based on the sign judgment principle of IRRAS^36^, both bands correspond to the out-of-plane vibration mode of N-O stretching. To the best of our knowledge, these two IR absorption bands of adsorbed NO have never been reported on single crystal rutile TiO_2_(110) surfaces.

To determine the NO adsorption states on reduced TiO_2_(110) at 0.01 L NO dosage, co-adsorption experiments of NO with CO or O_2_ were performed. Firstly, the reduced TiO_2_(110) surface was first pre-adsorbed with adequate CO (1 L), and then exposed to 0.01 L NO. In this case, as shown in Fig. 1b, only one negative band at 1600 cm^−1^ was observed for NO besides the vibrational bands (2186 cm^−1^ and 2190 cm^−1^) of adsorbed CO. Here, the 1600 cm^−1^ band has the same origin as the 1626 cm^−1^ band shown in Fig. 1a, the redshift results from the interaction between different molecules^37^. Since CO can only adsorb on Ti_5c_ sites rather than Vo sites^27^, the present 1600 (or 1626) cm^−1^ band is assigned to the stretching of NO adsorbed at empty Vo sites, while the absent 1751 cm^−1^ band is assigned to that of NO on Ti_5c_ sites. Secondly, the reduced bare TiO_2_(110) surface was pre-adsorbed with adequate O_2_ at 90 K and subsequently exposed to 0.01 L NO. In this case, both 1626 and 1751 cm^−1^ bands no longer occur in the spectra (See Figure S1 in the SI), while new 1874 cm^−1^ and 1743 cm^−1^ bands appear. The latter two bands correspond to the symmetric and asymmetric stretching vibrations of (NO)_2_ dimer adsorbed on surface Ti_5c_ sites^38^. Contrary to CO, saturatedly adsorbed O_2_ molecules occupy all the Vo sites and collect all the excess electrons of reduced TiO_2_(110) surface at low temperature^15^. The absence of 1626 cm^−1^ and 1751 cm^−1^ bands convincingly indicates that both bands should be related to the excess electrons.

DFT calculations were performed based on a 2 × 4 supercell to further clarify the adsorption configuration of NO and the role of polarons in molecular adsorption by means of comparing the calculated vibrational frequency (VF) of NO (see Table 1) with the experimental values. To verify the feasibility of the theoretical approach, we firstly calculated the distribution of polarons on the pristine TiO_2_(110) surface with one Vo. The lowest energy state among all possible polaron distributions corresponds to the case that two polarons are respectively localized at two nearest-neighboring Ti_6c_ sites in the subsurface from Vo (See Figure S2 in the SI), which is in good agreement with previous reports^39–41^. Furthermore, the calculation shows that the parallel spin alignment of two polarons and the antiparallel one are almost degenerate in energy, suggesting the weak exchange interaction between two polarons. This is also consistent with the recent calculation results^42^.

**Table 1:** Calculated vibrational frequency of adsorbed NO on TiO_2_(110) and their dependence on the location of polarons.

	Adsorption configuration	Number of excess electrons	Position of polaron	VF^DFT^ (cm^−1^)	VF^Exp^ (cm^−1^)
1	NO-Ti_5c_	0	—	1943	1870
2	NO-Vo (Figure 2a–c)	2	Vo/Ti_6c_	1636	1626
3		1	Vo	1640	
4		0	—	1965	
5	NO-Ti_5c_ & Vo (Figure 2d–f)	2	Ti_6c_/Ti_6c_	1942	
6		2	Ti_6c_/Ti_5c_	1770	1751

The experimental values are also listed in the last column for comparison.

**Figure 2:**
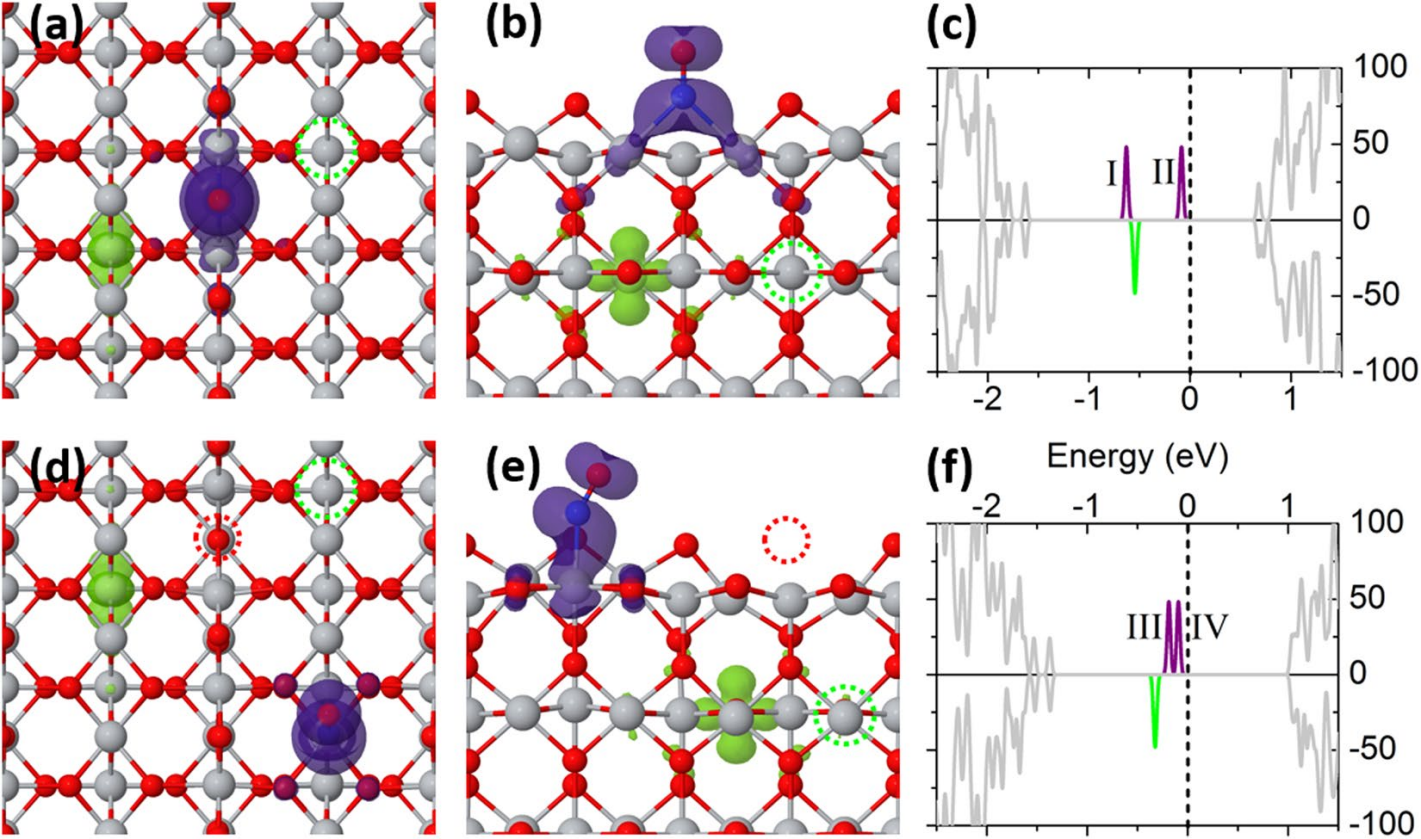
Structure, charge distribution of band-gap states (purple and green isosurfaces in the left and middle panels) and the corresponding density of states of NO-adsorbed TiO_2_(110) surfaces (in the right panel) for different NO adsorption configurations: (**a–c**) NO adsorbed at Vo; (**d–f**) NO adsorbed on Ti_5c_ of reduced surface with Vo (see the dashed red circles). Left panel: top view; middle panel: side view. For the DOS given in the right panel, the dashed vertical line denotes the Fermi level. The Ti:d-NO:p hybridized states near the Fermi level are shown by the purple peaks, and the spin-down gap state in green represents the polaron in the subsurface. The colorized states situated in the band gap are all rescaled by a factor of 2 for highlight.

When one NO molecule adsorbs at Vo sites and none of the surrounding Ti_5c_ cations is occupied, named configuration NO-Vo, as shown in Fig. 2a and b, the binding energy (E_b_) of NO molecules adsorbed at Vo is 1.09 eV. In this case, the calculated VF is 1636 cm^−1^ (2th entry), in good agreement with the experimental IR value (1626 cm^−1^) but markedly lower than that (1943 cm^−1^) of isolated NO adsorbed on defect-free surface (1st entry of Table 1) with E_b_ of 0.61 eV. The calculated density of states (DOS) is shown in Fig. 2c. Within the band gap, there are three states highlighted in different colors. The spin-up state lower in energy (peak I) consists of ${{\rm{Ti}}}_{{\rm{5c}}-{\rm{Vo}}}{:{\rm{d}}}_{{\rm{yz}}}/{{\rm{d}}}_{{{\rm{3z}}}^{{\rm{2}}}-{{\rm{r}}}^{{\rm{2}}}}$ and antibonding ${\text{NO}:{\rm{p}}}_{{\rm{y}}}^{\ast }$ orbitals, whereas the other one higher in energy (peak II) is composed of ${{\rm{Ti}}}_{\mathrm{5c}-\mathrm{Vo}}{:{\rm{d}}}_{{\rm{xz}}}/{{\rm{d}}}_{{\rm{xy}}}$ and antibonding ${\mathrm{NO}:{\rm{p}}}_{{\rm{x}}}^{\ast }$ orbitals. The spin-down state originates from the Ti_6c_-polaron state. Here, we found that the interaction between the Ti_6c_-polaron and the adsorbed NO is rather weak. Therefore, to get a clear view of the band structure, in the following discussions the Ti_6c_-polaron state is artificially located in the minority spin channel. Obviously, both hybridized ${\mathrm{NO}:{\rm{p}}}^{\ast }-{{\rm{Ti}}}_{{\rm{5c}}-{\rm{Vo}}}:{\rm{d}}$ states are occupied, in contrast to that of NO adsorbed on the defect-free TiO_2_(110) surface, where only one ${\text{NO}:{\rm{p}}}_{{\rm{y}}}^{\ast }$ molecule state is occupied within the band gap (See Figure S3 in the SI). The calculated band structure indicates that the NO adsorbed at Vo possesses an additional electron from the excess electrons of reduced TiO_2_(110) surface. Such result is further confirmed by the spatial charge distribution of the band gap states shown in Fig. 2a and b: there is only one Ti_6c_-polaron left but evident charge distribution mainly on NO is observed.

When the subsurface Ti_6c_-polaron was removed from the supercell, the calculated VF of NO at Vo is 1640 cm^−1^ (3rd entry), very similar to 1636 cm^−1^. Such result indicates that the subsurface Ti_6c_-polaron has little influence on the VF of NO adsorbed at Vo. However, if both excess electrons are removed, the calculated VF of NO at Vo dramatically shifts to 1965 cm^−1^ (4th entry), similar to that on the defect-free surface (1943 cm^−1^).

**Figure 3:**
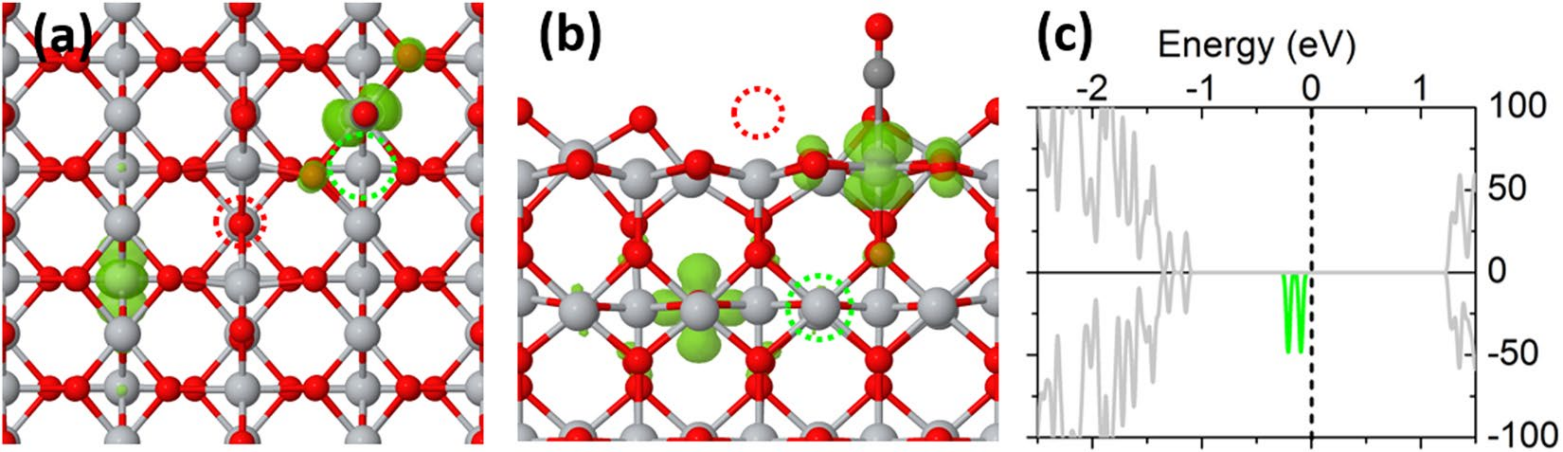
Structure, charge distribution of band-gap states (purple isosurface in the left and middle panels) and the corresponding DOS of CO-adsorbed TiO_2_(110) (in the right panel). Here, the CO adsorbs on the next-nearest neighboring Ti_5c_ of reduced surface from Vo (see the dashed red circles). Left panel: top view; middle panel: side view.

Besides the NO-Vo configuration, for the very low NO dosage at low enough temperature (e.g., 90 K in our experiments), another possible adsorption configuration is that one Ti_5c_ site near Vo site is occupied while the Vo remains unoccupied, named NO-Ti_5c_ & Vo. We found that the adsorption energy of NO on Ti_5c_ sites is hardly related to the distance between NO and Vo. Therefore we select the third-neighboring surface Ti_5c_ site relative to Vo to adsorb one NO, as shown in Fig. 2d. The structural optimization suggests a tilted orientation of the NO molecule, similar to that of NO adsorbed on the defect-free TiO_2_(110) surface^43, 44^. The DOS of such configuration with the lowest energy is shown in Fig. 3f, similar to that of NO-Vo configuration, where both hybridized ${\mathrm{NO}:{\rm{p}}}^{\ast }-{{\rm{Ti}}}_{\mathrm{5c}-\mathrm{Vo}}:{\rm{d}}$ states are occupied. The spatial charge distribution of the band gap states (Fig. 2d,e) shows that one Ti_6c_-polaron remains at the subsurface, and the other one transfers mainly to NO. The calculated VF is 1770 cm^−1^ (6th entry), in good agreement with our IR value in Fig. 1a (1750 cm^−1^). Therefore, we conclude that the IR-detected 1750 cm^−1^ band corresponds to the NO-Ti_5c_ & Vo adsorption configuration with one excess electron (polaron) involved. The corresponding E_b_ is 0.84 eV. However, if both Ti_6c_-polarons remain at the subsurface (5th entry), that is, neither polaron involves in the adsorption, the calculated VF of NO is 1942 cm^−1^ similar to the case of NO adsorbed on defect-free surface (1st entry of Table 1). Then the corresponding E_b_ is only 0.44 eV.

CO adsorption was also studied to further understand the role of polaron in molecular adsorption. STM and IR results revealed that CO preferentially adsorb on the next-neighboring Ti_5c_ site from Vo^31, 45^. For this configuration, the calculated electronic structure is shown in Fig. 3. It is found that CO adsorption induces one electron of subsurface Ti_6c_-polarons to transfer to the Ti_5c_ cation under CO, forming a surface Ti_5c_-polaron. Unlike NO adsorption, no electron transfers to the orbital state of CO. As a result, although the polaron involves in CO adsorption and thus the surface Ti_5c_-polaron is no longer degenerate with the subsurface Ti_6c_-polaron, the VF of CO has got little effect from the surface Ti_5c_-polaron (exp.: from 2188 cm^−1^ to 2178 cm^−1^ (Supplemental Information, Fig. S4; ref. 45); DFT: from 2178 cm^−1^ to 2155 cm^−1^). Such little effect is also embodied in the little enhancement of the binding energy, which is less than 0.05 eV.

Deskins *et al*. proposed that the electronegativity difference between the adsorbates and surfaces governs the surface-to-adsorbate charge transfer^29^. If the electronegativity of the adsorbates is greater than that of the substrates, the surface-to-adsorbate charge transfer will occur^29^. For NO and CO on reduced TiO_2_(110) surfaces in our experiments, the electronegativity of CO (~6.1 eV) is higher than that of NO (~4.5 eV)^29^, however, the surface-to-adsorbate charge transfer occurs only for NO on TiO_2_(110) rather than for CO on TiO_2_(110).

**Figure 4:**
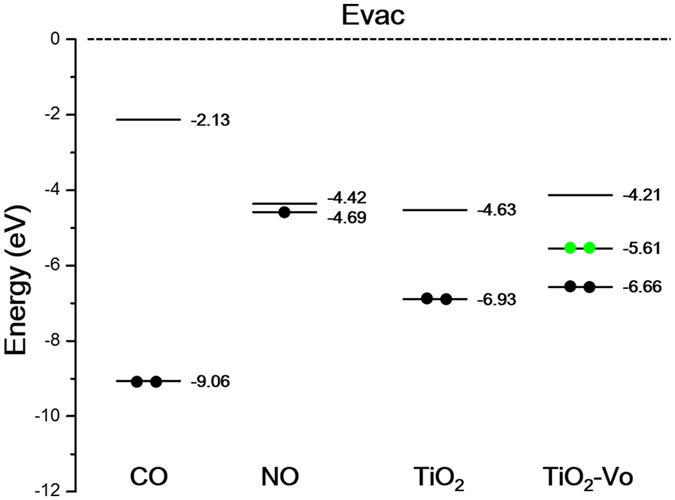
Calculated HOMO and LUMO of gas-phased CO and NO molecules, and band structures of the stoichiometric TiO_2_(110) surface and the reduced surface (TiO_2_-Vo) with respect to the vacuum energy level. Note that all the results are spin-degenerate expect for NO.

To understand the different scenarios of polarons in NO and CO adsorption, herein, we analyze the energy alignment between the molecular frontier orbitals and surface electronic states. The calculated highest occupied molecular orbital (HOMO), the lowest unoccupied molecular orbital (LUMO) of gas-phased NO and CO as well as the band structures near the band gap of TiO_2_(110) relative to the vacuum level are shown in Fig. 4. Due to the strong spin-splitting of NO orbitals, only the lower energy levels in the majority-spin channel are shown in this level range. Clearly, the LUMO of NO is at −4.42 eV, higher than the occupied valence band maximum level (−6.93 eV) of defect-free TiO_2_ by 2.51 eV, but only higher than the polaron state (−5.61 eV) of the reduced TiO_2_ by 1.19 eV. When NO adsorbs on the Ti_5c_ site of reduced TiO_2_(110) surface, the small energy level difference (1.19 eV) facilitates the hybridization between the NO LUMO and the polaron state, and the electron transfer occurs from the subsurface polaron to the Ti:3d-NO:2p hybrid orbital. Such involvement mode of polaron in NO adsorption causes the strong intramolecular modification and thus leads to the dramatic shift of NO vibrational frequency. In contrast, the LUMO of the free CO molecule is at −2.13 eV, much higher than that (−5.61 eV) of polaron states of TiO_2_(110) surface, so their hybridization hardly occurs, and the electron from TiO_2_ polarons cannot transfer to the molecular orbital of CO. That is, although the adsorption of CO induces the electron from the subsurface Ti_6c_ polaron state to the surface Ti_5c_ polaron state, such surrounding change only induces a weak shift of the CO vibrational frequency. Above analysis does not concern the barrier between two states for the charge transfer. According to our experiments, the barrier should be very low or be lowered by the coupling between adiabatic states proposed in recent DFT calculations for O_2_ adsorption on reduced TiO_2_(110) surfaces^46^.

## Conclusion

In summary, the polaron-involved molecular adsorption on reduced TiO_2_(110) surfaces was studied by IR spectroscopic and DFT approaches using NO and CO as probe molecules. Two typical scenarios were proposed: for NO adsorption, the subsurface polaron electron transfers directly to the empty molecular orbital due to the hybridization between the NO LUMO and the polaron state, the strong intramolecular modification causes the large shifts of the molecular vibrational frequency; for CO adsorption, the subsurface polaron only transfers to the surface Ti_5c_ cation underneath CO, the surrounding change results in a weak shift of the molecular vibrational frequency. These scenarios are determined by the energy-level matching between the polaron state and the LUMO of adsorbed molecules. Although such scenarios of polaron-involved molecular adsorption were obtained on rutile TiO_2_(110) surfaces, it is prospected that they should be also applicable to other reducible oxides.

## Methods

### Experimental details

The experiments were carried out in a state-of-the-art UHV-FTIRS system combining a vacuum FTIR spectrometer (Bruker, VERTEX 80 v) and a multichamber UHV system (PREVAC) with the base pressure better than 6 × 10^−11^ mbar, as described in detail previously^36^. The clean TiO_2_(110) surface was prepared by a sequence of Ar^+^ sputtering and annealing at 800 K in UHV conditions until a high-quality (1 × 1) low energy electron diffraction (LEED) pattern was obtained. During the preparation, Auger electron spectroscopy (AES) was used to monitor the clearance of the sample surface. After several times of such treatment, about 8% surface bridge oxygens were removed on the sample. The IR measurements were performed using infrared reflection absorption spectroscopy (IRRAS) mode with a fixed incidence angle of 80° respectively along $[1\bar{1}0]$ and [001] crystallographic directions. The IRRAS data were recorded at 90 K with 2048 scans at 4 cm^−1^ resolution. In experiments, high purity NO (99.9%) and CO (99.99%) were dosed via backfilling.

### Computational details

First-principles calculations were performed using the Vienna ab-initio simulation package (VASP)^47^ with a cut-off energy of 500 eV for the basis set. Γ-point is used for Brillouin zone sampling. The projector-augmented wave method (PAW)^48^ with the PBE type^49^ exchange–correlation potentials was adopted. To correctly model the localization of excess electrons in rutile TiO_2_^17^, the DFT + U method with U_Ti:3d_ = 4.2 eV^50^ is employed. Furthermore, a van der Waals dispersion correction based on the D2 method is included in the calculation^34, 51^. The vibrational frequencies are derived from Hessian matrix calculated by finite-displacement method. To model the TiO_2_(110) surface, the experimentally determined lattice parameters of bulk rutile TiO_2_, a = 4.594 Å and c/a = 2.959^52^, were used to build a slab including four tri-layers and a vacuum layer with a thickness of 15 Å. A supercell with a p(4 × 2) geometry along $[001]$ and $[1\bar{1}0]$ directions, respectively, was employed to perform the calculations. The atomic positions of top three tri-layers were optimized until the forces are less than 0.02 eV/Å, while the bottom tri-layer were fixed at bulk positions and were terminated by pseudo-hydrogen atoms^53, 54^. To manipulate the location of polaron, a preliminary lattice-distortion approach^40^ was employed, where the bonds between the targeted Ti cation and its neighboring O anion were initially stretched by ~0.1 Å before the structural optimization. During the calculation, removing the subsurface Ti_6c_-polaron from the supercell was realized by altering the number of valence electrons.

## Electronic supplementary material

Supplemental Information
