# Supplementary material for: Scenarios of polaron-involved molecular adsorption on reduced TiO2(110) surfaces
Source: Sci Rep. 2017 Jul 21;7:6148. doi: 10.1038/s41598-017-06557-6 (PMC5522416; doi:10.1038/s41598-017-06557-6)
Supplement: Supplementary file 1 — Supplemental Information [file 41598_2017_6557_MOESM1_ESM.doc]

Supplemental Material for

**Scenarios of polaron-involved molecular adsorption on reduced TiO_2_(110) surfaces**

Yunjun Cao, Min Yu, Shandong Qi, Shiming Huang, Tingting Wang, Mingchun Xu*, Shujun Hu*, and Shishen Yan

*School of Physics, State Key Laboratory of Crystal Materials, Shandong University, 27 Shanda*

*Nanlu, Jinan, Shandong 250100, P. R. China*

**Email M.C.X.: xumingchun@sdu.edu.cn*

**Email S.J.H.: hushujun@sdu.edu.cn*

**Supplemental Figures**

**Figure S1** Non-polarized IRRA spectra of NO adsorbed on 2 L O_2_ pre-adsorbed TiO_2_(110) surface at 90 K. In this case, at very low NO dosage (0.01 L), the negative absorption bands at 1750 cm^-1^ and 1625 cm^-1^ found on reduced TiO_2_(110) surface (Fig. 1a) at both incidence plane no longer appear. Instead, the bands at 1874 cm^-1^ and 1743 cm^-1^ occur, such bands correspond to the symmetric and asymmetric stretching vibrations of (NO)_2_ dimer adsorbed on surface Ti_5c_ sites. The dimer configuration is consistent with our previous reports on defect-free TiO_2_(110) surface ^1^.


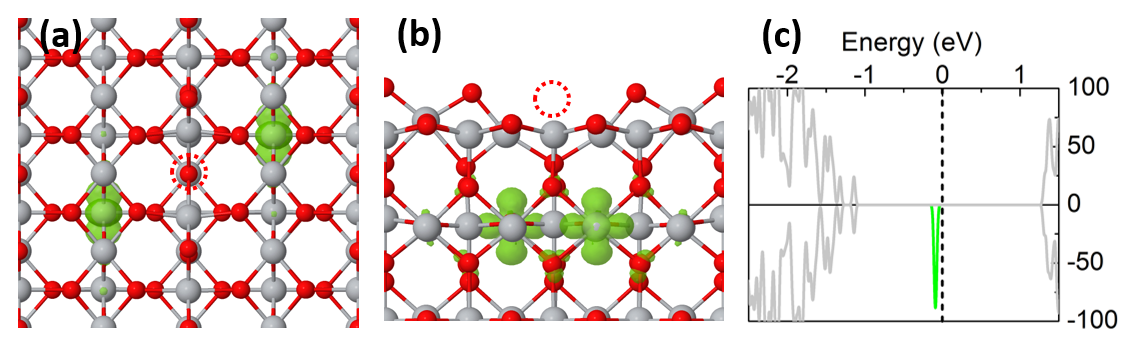


**Figure S2** (a) and (b) Charge distribution of the polaron states (Left panel: top view; middle panel: side view) of the bare TiO_2_(110) surface with one Vo and (c) the calculated DOS. Vo is indicated by the dashed red circle. Two polarons are degenerate in energy.


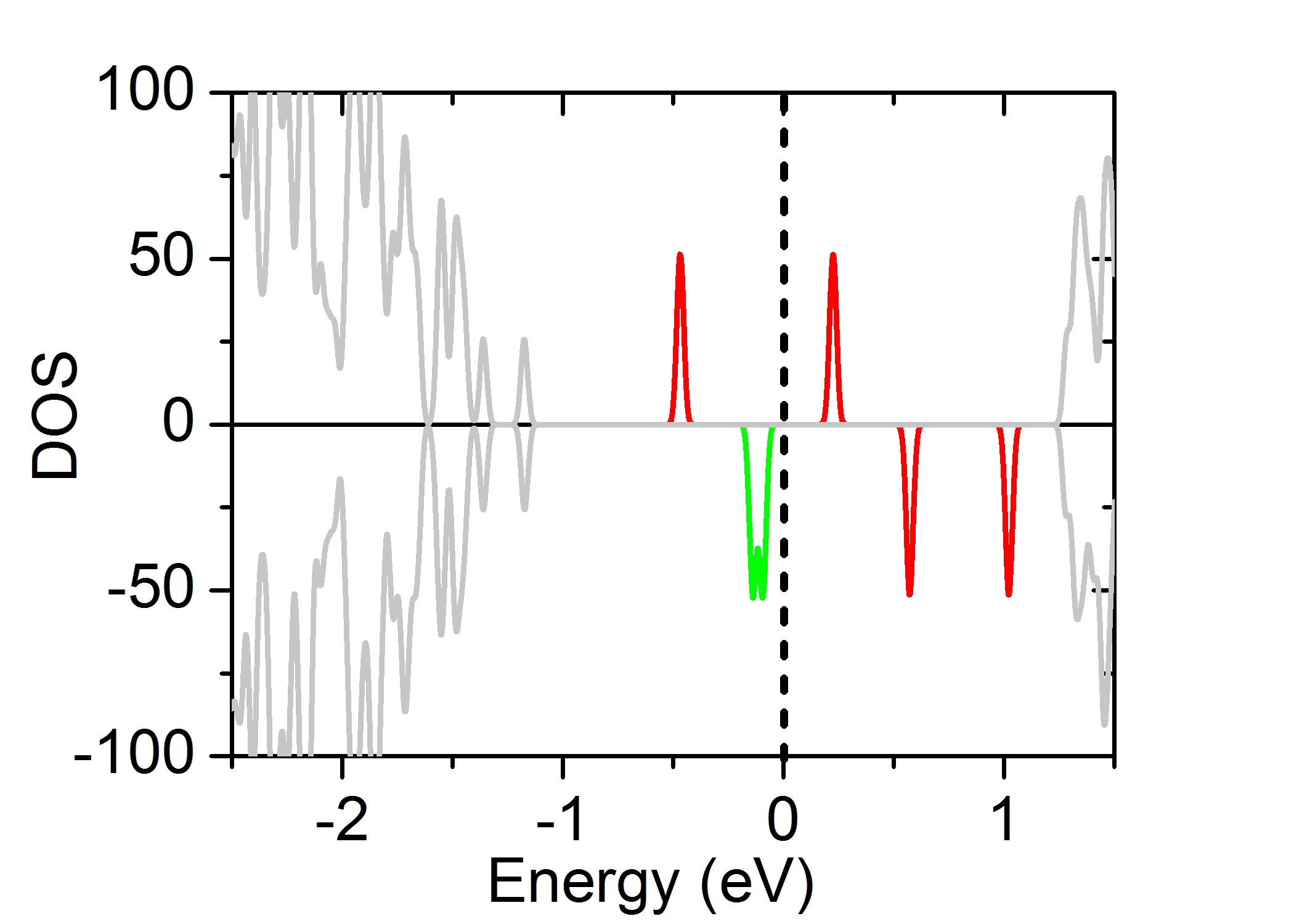


**Figure S3** Density of states of NO-adsorbed TiO_2_(110) with one Vo, where the two polaron states (green peaks) are fixed at Ti_6c_ sites of the subsurface. Red peaks denote the molecular orbitals of NO near the Fermi level.

**Figure S4** Non-polarized IRRA spectra of 2 L CO adsorbed on reduced TiO_2_(110) surface and 2 L O_2_ pre-adsorbed TiO_2_(110) surface at 100 K, respectively.

References:

1 Xu, *M. et a*l. NO adsorption and reaction on single crystal rutile TiO2(110) surfaces studied using UHV-FTIRS*. Phys Chem Chem Ph*y**s** 16, 14682-14687 (2014).
